# Chemical and Physical Characterisation of Macroaggregated Human Serum Albumin: Strength and Specificity of Bonds with ^99m^Tc and ^68^Ga

**DOI:** 10.3390/molecules27020404

**Published:** 2022-01-09

**Authors:** Letizia Canziani, Manuela Marenco, Giorgio Cavenaghi, Giulia Manfrinato, Angelo Taglietti, Alessandro Girella, Carlo Aprile, Giovanna Pepe, Lorenzo Lodola

**Affiliations:** 1 Fondazione IRCCS Policlinico San Matteo, Nuclear Medicine Unit, 27100 Pavia, Italy; g.cavenaghi@smatteo.pv.it (G.C.); g.manfrinato@smatteo.pv.it (G.M.); carlo.aprile@icloud.com (C.A.); gi.pepe@smatteo.pv.it (G.P.); l.lodola@smatteo.pv.it (L.L.); 2 Chemistry Department, University of Pavia, 27100 Pavia, Italy; angelo.taglietti@unipv.it (A.T.); alessandro.girella@unipv.it (A.G.)

**Keywords:** radiopharmaceutical, PET, SPECT, macroaggregated, human serum albumin, ^99m^Tc, ^68^Ga

## Abstract

Background: Macroaggregated human serum albumin (MAA) properties are widely used in nuclear medicine, labelled with ^99m^Tc. The aim of this study is to improve the knowledge about the morphology, size, dimension and physical–chemical characteristics of MAA and their bond with ^99m^Tc and ^68^Ga. Methods: Commercial kits of MAA (Pulmocis^®^) were used. Characterisation through experiments based on SEM, DLS and Stokes’ Law were carried out. In vitro experiments for Langmuir isotherms and pH studies on radiolabelling were performed and the stability of the radiometal complex was verified through competition reactions. Results: The study settles the MAA dimension within the range 43–51 μm. The Langmuir isotherm reveals for [^99m^Tc]MAA: Bmax (46.32), h (2.36); for [^68^Ga]MAA: Bmax (44.54), h (0.893). Dual labelling reveals that MAA does not discriminate different radioisotopes. Experiments on pH placed the optimal pH for labelling with ^99m^Tc at 6. Conclusion: Radiolabelling of MAA is possible with high efficiency. The nondiscriminatory MAA bonds make this drug suitable for radiolabelling with different radioisotopes or for dual labelling. This finding illustrates the need to continue investigating MAA chemical and physical characteristics to allow for secure labelling with different isotopes.

## 1. Introduction

Macroaggregated albumin (MAA) labelled with technetium-99 m (^99m^Tc) is one of the more common forms of human serum albumin (HSA) microparticles used in nuclear medicine [1]. MAAs are formed by heat-denaturing albumin in a bulk solution containing a reducing agent such as SnCl_2_·2H_2_O, controlling the size (median 50 µm) and dispersion of the preparation particles. Owing to their dimension, after injection, MAAs are trapped in the first precapillary vessel encountered, which later undergoes enzymatic digestion.

Introduced in nuclear medicine armamentarium during the seventies, ^99m^Tc labelled MAAs were predominately used for lung perfusion imaging. The time–activity curves in human lungs have a biexponential shape with components of effective half-lives 0.88 and 4.56 h [2].

In the last two decades, a change in patterns of Tc-MAA has been observed with a shift from lung scan for pulmonary embolism because of extensive use of CT angiography, towards other uses such as ROLL (Radio Occult Lesion Localisation), surrogate particle of yttrium microspheres for the assessment of arterial supply and dosimetry, and for a wide series of less common applications [3,4,5,6,7,8].

More recently, there was a renewed interest for this kind of particle labelled with the positron emitter ^68^Ga (t1/2 = 67.71 min, positron emission 89%), conveniently obtained from a ^68^Ge/^68^Ga generator [9].

The advantages of PET detection over SPECT are significant as better spatial resolution and the possibility to quantify the tissue uptake (SUV) can be achieved, which allows clinicians to predict intraoperative detectability.

Many studies have already been carried out, showing a better diagnostic performance of Ga over Tc label [10,11,12].

Usually a prewash of MAA in commercial kit resulted in a higher gallium labelling efficacy, likely due to the removal of the free HSA contained therein as excipient [13].

This step compromises the sterility of the solution, thus making subsequent drug administration to patients problematic. Nevertheless, a direct labelling without any prewash or purification step has been achieved with a satisfactory labelling yield, thus allowing at the same time the classical Technetium labelling and the optical agent Indocyanine Green binding [14]. One can postulate that MAA can offer multiple binding sites for reason of its high volume and surface.

For this reason, research interest is now increasingly focused on the possibility of labelling commercial kits routinely used with this isotope in order to extend diagnostic accuracy, surgical outcome, follow-up and even therapeutic horizons.

However the nature of the complexation or the oxidation state of Tc has not been fully elucidated [15].

An early study by Steigman suggested that reduced Tc links to SH groups; however, this hypothesis reported in a congress abstract was neither validated or developed in a later full paper [16].

Similarly, the chemical nature of Ga binding to HSA particles has not been fully elucidated. Two hypothetical mechanisms have been postulated, one involves the absorption of insoluble hydrolysed gallium hydroxide on the surface of MAA while the other suggests a specific interaction of ionic Ga with a protein ion pair [17].

In addition, information about the physical–chemical characteristics of MAA is scarce; therefore, there is a need to expand our knowledge of the bond between MAA and radionuclides, their strength and specificity. For this reason, we studied MAA morphology and dimensional distribution through experiments based on SEM, DLS and Stokes’ Law. Langmuir isotherms made it possible to find the maximum concentration of complexable radiometal. The bond strength and specificity were indirectly investigated by changing the starting labelling conditions, through the simultaneous presence of the two isotopes and competitive reaction with a highly competitive ligand, DMSA. Additionally, the pH effect on radiolabelling with ^99m^Tc was tested.

Thus, it is mandatory to increase the knowledge of the bond between MAA and the isotopes to allow a possible replacement for this pharmaceutical.

## 2. Materials and Methods

### 2.1. Materials

All the pharmaceuticals used in this study were already commercialised and authorised for clinical use. Macroaggregate of human serum albumin colloidal particles is a radiopharmaceutical that is commercially available as Pulmocis^®^ (Curium—CIS bio international, F-Gif sur Yvette, Paris, France). Active ingredient: macroaggregated human serum albumin colloidal particles 2.0 mg, with a diameter up to 150 µm and a number of particles per vial ranging between 2 × 10^6^ and 4.5 × 10^6^. Excipients: stannous (II) chloride dihydrate, human albumin, E941, E512, sodium chloride, sodium caprylate. In this article, the Pulmocis^®^ kit is generically referred to as MAA.

Human serum albumin is a radiopharmaceutical commercially available as Vasculocis^®^, containing 10 mg of human serum albumin, stannous chloride dihydrate, hydrochloric acid and sodium chloride, under nitrogen atmosphere (Curium—CIS bio international, F-Gif sur Yvette, Paris, France). In this article, Vasculocis^®^ is generically referred to as HSA.

Dimercaptosuccinic acid (DMSA) is a radiopharmaceutical commercially available as Renocis^®^, containing 1 mg of dimercaptosuccinic acid, stannous chloride dihydrate, inositol and ascorbic acid. In this article, the Renocis^®^ kit is generically referred to as DMSA (Curium—CIS bio international, F-Gif sur Yvette, Paris, France).

All kits were labelled with sodium pertechnetate [^99m^TcO_4_^−^], obtained from ^99^Mo/^99m^Tc Tekcis^®^ generator (Curium Cis-Bio, Gif-sur-Yvette, Paris, France) and sodium chloride for injection (0.9%). To perform ^68^Ga labelling, ^68^Ge/^68^Ga generator (1.1 GBq TiO_2_-based GalliaPharm^®^ Eckert-Ziegler Isotope Products, Berlin, Germany) was eluted with 8 mL 0.1 N HCl (Eckert-Ziegler); 0.75 mL 0.1 N NaOH/phosphate buffer (1 mL) was added to this 0.1 N HCl solution of [^68^Ga] Ga-chloride. Final pH ranged between 4.0 and 4.5.

All chemicals were manufactured by TraceSELECT–UltraPURE from ABX Radeberg, Germany.

### 2.2. Methods

#### 2.2.1. Quality Controls and Labelling Yield

Quality controls (QC) were performed to verify the labelling yield of [^99m^Tc]Tc-MAA and [^68^Ga]Ga-MAA. The percentage of free ^99m^Tc was evaluated by thin-layer chromatography, using ITLC-SG (Varian, Folson, East Grand Forks, MN, USA) as a stationary phase (10 cm long and 2 cm wide) and CH_3_OH:H_2_O 85:15 as a mobile phase. A 10 μL spot of solution containing the sample was applied to a strip roughly 1.5 cm from the bottom edge. Labelled MAA remains at the point of application, while free ^99m^Tc pertechnetate migrates with the solvent front. The percentage of free ^68^Ga was assessed in the same way, using 0.1 M tribasic-citrate solution, adjusted to pH 6 with HCl, as a mobile phase. MAA labelled with ^68^Ga remains at the point of application, while free ^68^Ga migrates with the solvent front. The percentage of ^99m^Tc bond to HSA, a Pulmocis excipient, was evaluated by filtration with a polycarbonate membrane filter 13 to 25 mm in diameter, 10 µm thick and pores 3 µm in diameter. Two-tenths millilitre of solution was placed on the membrane, which was then rinsed with 20 mL of 0.9% sodium chloride solution. The eluate was collected in a syringe. Labelled MAA remains in the membrane, while [^99m^Tc]Tc-HSA and free ^99m^Tc pertechnetate are collected in the syringe. The activity of the membrane was determined with a 3” × 3” NaI(Tl) pinhole 16 × 40 mm gamma counter (Raytest, Straubenhardt, Germany). The solution collected in the syringe was evaluated by thin-layer chromatography using ITLC-SG (Varian, Folson, East Grand Forks, MN, USA) as a stationary phase (10 cm long and 2 cm wide) and CH_3_OH:H_2_O 85:15 as a mobile phase. Labelled HSA remains at the point of application, while free ^99m^Tc pertechnetate migrates with the solvent front; ^99m^Tc and ^68^Ga activity was determined with a 3” × 3” NaI(Tl) pinhole 16 × 40 mm gamma counter (Raytest, Straubenhardt, Germany).

MAA characterisation underwent the following procedures:

#### 2.2.2. Study of MAA Dimension under 10 μm

To evaluate the fraction of MAA particles under 10 μm, a dynamic light scattering (DLS) Zetasizer Nano ZS90 Malvern Instrument was used. Three samples were analysed:

Sample 1: A commercial kit of MAA was dissolved in 4 mL of deionised water;
Sample 2: A commercial kit of MAA was dissolved in 12 mL of deionised water;
Sample 3: A commercial kit of MAA was dissolved in 12 mL of deionised water and underwent a heat treatment at 40 °C for 20 min.

#### 2.2.3. Study of MAA Dimension above 10 μm

The Andreasen method based on the Stokes’ Law was useful for determining the average size and the distribution pattern of MAA particles. The Stokes’ diameter distribution of the particles is deduced from the study of the particle concentration changes occurring within a settling suspension. A physiological solution, previously degassed with He_2_, was placed in a vertical glass tube of 40 cm length and 1 cm diameter, in front of an Infinia gamma camera detector (General Electric) equipped with an HR collimator. At t = 0, 0.1 mL of a solution composed of 2.0 mg of MAA in 4 mL deionised water, and a final activity of 30 MBq of ^99m^Tc, was placed on top of the tube. The measurements were performed for 10 min.

#### 2.2.4. MAA Morphological Study with SEM

A lyophilised commercial kit was analysed with a scanning electron microscope (SEM) by Zeiss, model EVO MA10, with 2500× to 25,000× magnification.

#### 2.2.5. [^99m^Tc]Tc-MAA Binding Affinity Studies

Samples containing 20 µg of MAA in 1 mL of deionised water were incubated with increasing concentration of ^99m^Tc (125, 350, 600, 1500, 2500, 3500 MBq that are equivalent to 10, 30, 50, 100, 200, 300 nmol ^99m^Tc/mg MAA). The incubation time was fixed at 20 min and the reaction was carried out at room temperature. All measurements were carried out under the same counting position, calibrated by standard source, correcting the data for background and decay. All experiments were carried out in duplicate and repeated three times. The uptake measurements were expressed in nmol ^99m^Tc bound/mg MAA.

#### 2.2.6. [^68^Ga]Ga-MAA Binding Affinity Studies

The procedure described above was replicated to evaluate the binding affinity of MAA with ^68^Ga. At the fixed incubation times of 20 min, MAA samples were incubated with increasing concentration of ^68^Ga (20, 40, 70, 120, 250, 350, 750 KBq that are equivalent to 3, 6, 12, 20, 40, 60, 120 nmol ^68^Ga/mg MAA). The reaction was carried out at 40 °C. The uptake measurements were expressed in nmol ^68^Ga bound/mg MAA.

#### 2.2.7. Competition Evaluation between ^68^Ga and ^99m^Tc for MAA Labelling

To verify the ^99m^Tc and ^68^Ga competition for MAA binding sites, a commercial kit of MAA was dissolved in 10 mL of saline solution; 10 nmol of ^99m^Tc (115 MBq) and 15 nmol of ^68^Ga (100 KBq) were added to 1 mL of this solution and incubated for 20 min at room temperature. The measurement of the ^99m^Tc vs. ^68^Ga bound was performed after 1 and 3 h.

#### 2.2.8. Effects of pH on [^99m^Tc]Tc-MAA Labelling

Two methods were used to evaluate the effect of pH on the [^99m^Tc]Tc-MAA radiolabelling. A commercial kit of MAA was first dissolved in 10 mL of saline solution, then 6 vials were prepared with 100 µL of this suspension and finally, 300 KBq of ^99m^Tc was added in each vial.

Method 1: The solution was incubated at pH 6 for 20 min at room temperature. At the end of incubation, the pH of 2 vials was adjusted to pH 3 and pH 10 with HCl 5 M and NaOH 2 M, respectively.
Method 2: The pH of 3 vials was immediately adjusted to reach a final pH value of 3, 6 or 10 with HCl 5 M and NaOH 2 M and incubated for 20 min at room temperature.

#### 2.2.9. [^99m^Tc]Tc-HSA Stability Test via DMSA Competition

Two methods were used to evaluate the stability of [^99m^Tc]Tc-HSA through competition with DMSA. DMSA was chosen as a highly competitive ligand, able to compete for ^99m^Tc sequestration. A large excess of DMSA was used.

Method 1: Commercial kits of DMSA and HSA previously dissolved in 4 mL of deionised water were mixed; 200 MBq of 99mTc were added and the resulting solution was incubated at pH 6 for 20 min at room temperature.
Method 2: A commercial kit of HSA was dissolved in 4 mL of deionised water and 200 MBq of 99mTc were added for incubation at pH 6, for 20 min at room temperature. A solution of the DMSA commercial kit was then dissolved in 4 mL of deionised water.

#### 2.2.10. [^99m^Tc]Tc-MAA Stability Test via DMSA Competition

The procedure described above was replicated to verify the stability of [^99m^Tc]Tc-MAA.

### 2.3. Mathematical Analysis

The Andreasen method was applied to Stokes’ Law to define the dimensional distribution of MAA particles.

For Stokes’ Law, the resistance exerted by a fluid on the movement of a particle, viscous friction, depends on the radius of the particle, velocity of the particle and viscosity of the fluid [18] (Equation (1)):

(1) $$v=\frac{2}{9}*g*r^{2}*\;\frac{\left(Ds-Df\right)}{u}\;$$

where *u* = physiological solution viscosity 1.014 cP, *D_f_* = physiological solution density 1.0046 g/mL, *D_s_* = density of human serum albumin 1.288 g/mL, *g* = acceleration of the force of gravity 9.81 m/s^2^ and *v* = speed (s/t).

Thus, the radiolabelled particulate will settle at different rates according to Stokes’ Law, depending on its radius (Equation (2)):

(2) $$r=\sqrt{\frac{\left(v*9*u\right)}{\left(Ds-Df\right)*g*2}}$$

A further step was to evaluate the amount of ^99m^Tc and ^68^Ga binding to each macroaggregated particle, and hence also each human serum albumin molecule, within a single MAA.

For the Langmuir isotherm, the amount of bound ^99m^Tc and ^68^Ga was plotted against total ^99m^Tc/mg of MAA and ^68^Ga/mg of MAA and the data were fitted by nonlinear fitting, as the one site-specific binding with the Hill slope equation (Equation (1)) in GraphPad Prism 5 (GraphPad Software, San Diego, CA, USA)

(3) $$Y=Bmax\;\frac{X^{h}}{Kd^{h}+X^{h}}$$

where *Y* is the concentration of bound radioligands, Bmax is the maximum of specific binding sites, *X* is the total concentration of radioligands, *Kd* is the ligand concentration that binds to half the binding sites at equilibrium and *h* is the Hill slope. If *h* equals 1.0, then binding with no cooperativity to one site occurred; when *h* is greater than 1.0, then multiple binding sites with positive cooperativity is implied. The Hill slope is less than 1.0 when there are multiple binding sites with different affinities for ligands or when there is negative cooperativity.

From the *Kd* value, the number of atoms bound, at equilibrium, to each single MAA molecule was calculated. The maximum specific binding provided from *Bmax* made it possible to determine the number of atoms specifically bound to each MAA particle.

The results obtained can be directly related to patients, because a proportion was made between the amount of MAA, ^68^Ga and ^99m^Tc used for experiments and the activity routinely administered.

## 3. Results

### 3.1. Quality Controls and Labelling Yield

**Figure 1:**
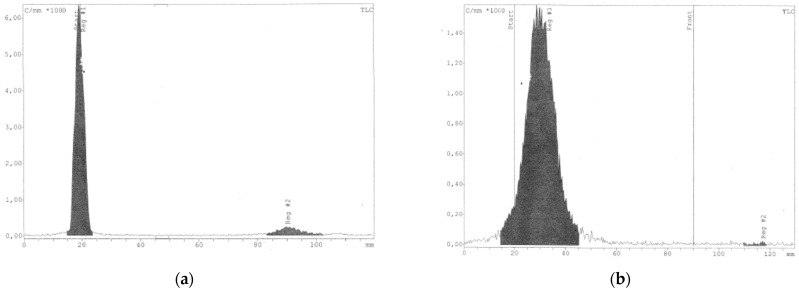
Quality controls (QC) performed to verify the labelling yield of ^99m^Tc bound/unbound to MAA (**b**) and ^68^Ga bound/unbound to MAA (**a**). Labelled MAA remains at the point of application, while free isotope migrates with the solvent front (TLC-SG; CH_3_OH:H_2_O 85:15 and 0.1 M tribasic-citrate solution for Tc and Ga, respectively).

Quality controls (QC) were necessary for every calculus. [^99m^Tc]Tc-MAA show a narrow peak with a RF of 0.2, while free ^99m^Tc show a RF of 0.9. [^68^Ga]Ga-MAA show a RF of 0.3–0.4, while free ^68^Ga show a RF of 0.95 (Figure 1). Table 1 prove that the radiolabelling yield of Pulmocis with ^99m^Tc and ^68^Ga is different (95% vs. 83%), probably due to the present of HSA as excipient.

### 3.2. Study of MAA Dimension under 10 μm

The dimensions of the three samples tested were >1 μm.

Sample 1: Average size was >10 μm.
Sample 2: Obtained from a 1:3 dilution of sample 1, its average size was attested at 3 μm and the PDI value was 0.183, meaning that 3 μm is the average size of all particles within the dimensional fraction <10 μm.
Sample 3: Heated at 40 °C for 20 min, results show the number of the MAA particles with size >10 μm reduced by about one-third after the heating treatment.

### 3.3. Study of MAA Dimension above 10 μm and Size Distribution of the Particles

The results settled MAA dimensions within the range 43–51 μm (Table 2). According to these findings, the number of human serum albumin molecules within each MAA dimensional fraction was derived.

### 3.4. MAA Morphological Study with SEM Microscopy

**Figure 2:**
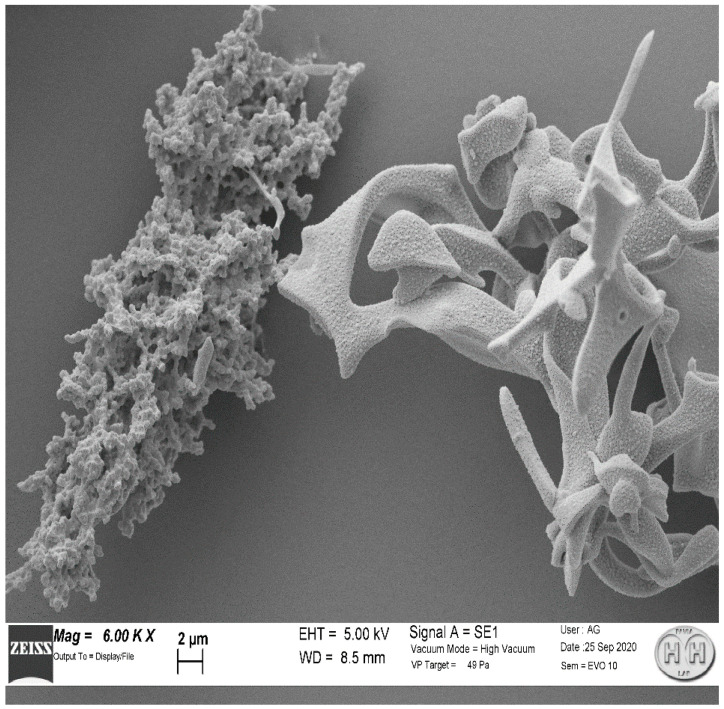
MAA particles showed two main structures (6.00 K ×).

SEM microscopy was helpful for investigating the morphology of MAA particles, SEM images proved that MAA are constituted by two different structures with distinct morphology present in approximately equal measure (Figure 2).

**Figure 3:**
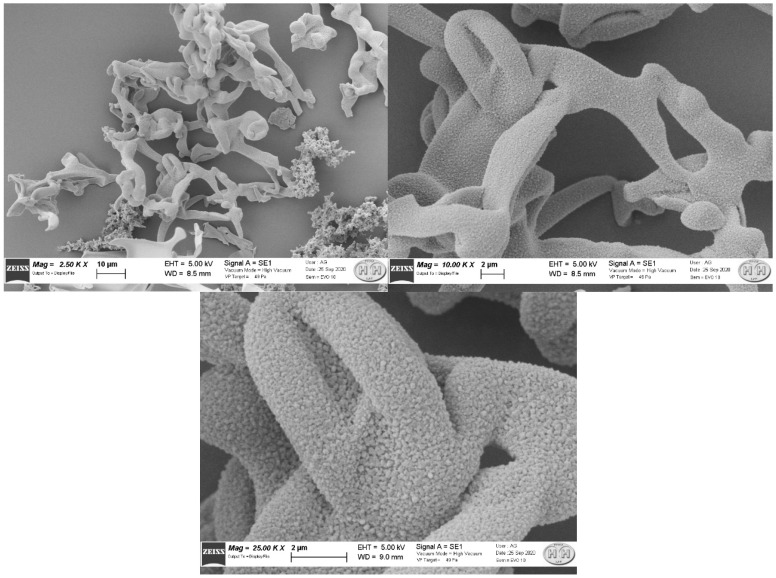
Successive enlargements of the first type of MAA structure (2.50 K ×, 10.00 K ×, 25.00 K ×).

**Figure 4:**
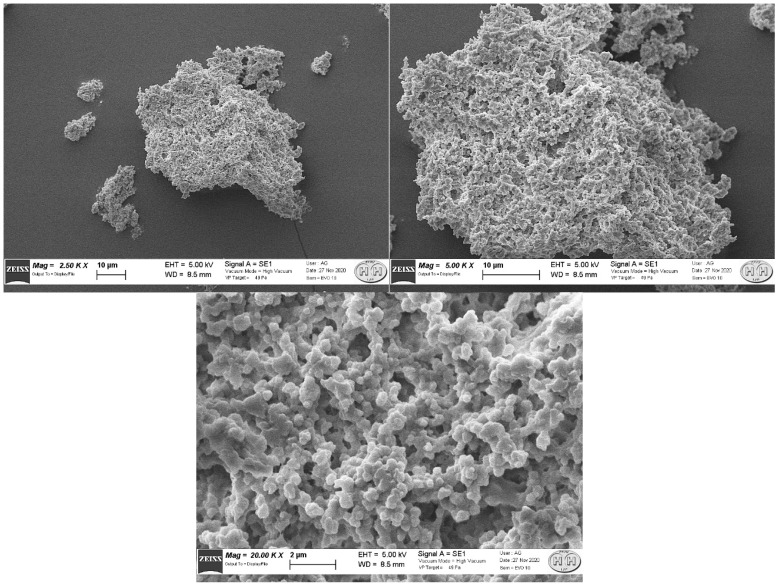
Successive enlargements of the second type of structure (2.50 K ×, 5.00 K ×, 20.00 K ×).

Figure 3 and Figure 4 represent three enlargements of the two structures, representative of the sample. The two structures presented complex shapes, with a surface area significantly larger than a spherical shape of equivalent diameter. These results are compatible with the MAA particle dimensions determined with the above-described experiments.

### 3.5. [^99m^Tc]Tc-MAA and [^68^Ga]Ga-MAA Binding Affinity Studies

Through the Langmuir isotherm, the following data are obtained: [^99m^Tc]Tc-MAA: Kd 65.54, Bmax 46.32, h 2.36 with R^2^ 0.9836; [^68^Ga]Ga-MAA: Kd 77.47, Bmax 44.54, h 0.8935 with a R^2^ 0.9968 (Figure 4).

The number of isotopes bound at equilibrium and specifically bound is calculated from the Langmuir isotherm result, normalised to the number of MAA and then to the number of HSA that form MAA (Table 3).

### 3.6. ^68^Ga and ^99m^Tc Competition for MAA

As shown in Table 4, at first the amount of ^68^Ga bound to MAA was greater than ^99m^Tc, but after an incubation of 3 h, at ambient T and pH 6, the molar fraction of bound/unbound atoms was reversed, with a significant increase of ^99m^Tc bound. The comparison was made between the ^99m^Tc concentration bound to MAA in presence of ^68^Ga, using the concentration that is usually used in nuclear medicine.

### 3.7. Effects of pH on [^99m^Tc]Tc-MAA Labelling 

The pH effect on labelling was tested by changing the pH either after radiolabelling (Method 1) or simultaneously (Method 2). When changes occurred within the standard labelling procedure (Method 2), the molar fraction of ^99m^Tc bound to MAA was not affected. Instead, when changes occurred after the standard labelling procedure (Method 1) at pH 3 the labelling was lower, while at pH 10 the conjugation did not occur (Table 5). The results obtained with both Method 1 and Method 2 placed the optimal pH for labelling at 6.

### 3.8. HSA and MAA Competition with DMSA for ^99m^Tc Bound Stability

Regarding HSA, the [^99m^Tc]Tc-DMSA complex formed with Method 1 was more stable than the [^99m^Tc]Tc-HSA complex (0.087 vs. 0.013, respectively). In Method 2, DMSA was able to transchelate about 30% of the ^99m^Tc previously bound to HSA (0.029 vs. 0.071), as shown in Table 6.

The same occurred for competition with MAA: [^99m^Tc]Tc-DMSA complex formed with Method 1 is more stable than the [^99m^Tc]Tc-MAA complex (0.070 vs. 0.029, respectively). In Method 2, DMSA was able to transchelate about 30% of the ^99m^Tc previously bound to MAA (0.079 vs. 0.021), as shown in Table 7.

## 4. Discussion

To our knowledge, this aspect of two morphologically different MAA populations at SEM scale has not been previously described. We do not know how much the different shape can interfere with the physicochemical properties and if this aspect is related only to the preparation we investigated or if it is a common characteristic of any commercial kit. The SEM images show that one structure is consistent with those reported by other studies [19,20].

Both structures present a highly wrinkled surface. It is recognised that structures with greater roughness have a greater probability of presenting very different binding sites [21]. Thus, we can note that MAA from Pulmocis^®^ present very complex shapes, therefore, with considerably larger surfaces than a spherical shape of equivalent diameter, which is the case instead for nanocolloids of human serum albumin (NC), which have a spherical or ellipsoidal structure [14], thus enhancing the reactivity properties. Although it is impossible to separate the two structures in order to carry out further experiments, the structures with a greater amount of wrinkled surface (Figure 4) could probably provide better radiolabelling properties than the other type (Figure 3).

The measurements through DLS helped to verify whether the dimensions of MAA really attest in the order of micrometres, as well as if the heating process affects the dimensional distribution. The 40 °C heating incubation of Pulmocis^®^ leads to the partial loss of MAA structure and to a dimensional decrease of one-third, confirming the results of Persico et al. [14]. Referring to the SPC of other pharmaceutical preparations, the heat treatment could be necessary to labelling MAA with ^68^Ga, causing the fragmentation of the MAA and compromising several in vivo diagnostic applications of these compounds. Thus, it is therefore important to focus on alternative ways for providing energy to the system.

QC after three micron filtration shows that more than 95% of ^99m^Tc is bound to MAA, 4.09% to HSA and 0.78% is free, while only 83% of ^68^Ga is bound to MAA, compared to 15.66% to HSA and 1.11% ascribable to the free isotope (Table 1). Commercial MAA kits contain a variable amount of added HSA; in the specific case of Pulmocis, the range is 8.7 mg of HSA in comparison to 2 mg of HSA as MAA. The explanation for the higher amount of ^68^Ga bound to the albumin fraction might be not univocal; we can hypothesize the affinity of gallium can be attributed to the huge amount of HSA in the Pulmocis^®^ kit.

These impurities could potentially affect the in vivo PET performance, lowering the lung-to-liver ratio. However, additional pre- or post-labelling purification steps increase the risk of microbial contamination and the dose to the operator. From this point of view, the use of a fully automated GMP compliant synthesiser could obviate these limits [22]. In the final calculation we subtracted the percentage of isotopes bound to HSA.

To evaluate the size of particles larger than 10 μm, the experiment of the Andreasen method based on Stokes’ Law was used. The procedure makes it possible to establish the number of particles for each dimensional class. The results, reported in Table 2, show that the largest percentage is for particle sizes between 43 and 51 μm.

To characterise the bond characteristic between isotopes and MAA, an attempt was made to evaluate the kinetics of radiolabelling of MAA with ^99m^Tc. The speed of radiolabelling was too rapid to detect the variation of MAA labelled (95% labelling in less than 60 s); it should be noted that the speed increase from HSA to NC [23] to MAA is probably due to the greater reaction surface.

**Figure 5:**
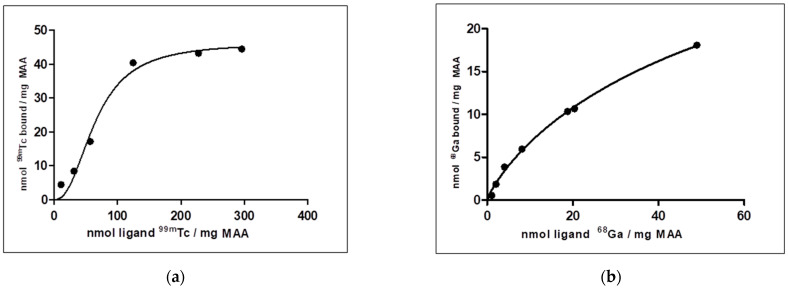
Saturation binding experiments (one site-specific binding determined by the Hill slope equation) for binding affinity between MAA and ^99m^Tc (**a**) and ^68^Ga (**b**).

Owing to studies of binding affinities, it was determined that MAA can bind both ^99m^Tc and ^68^Ga, and both produced a sigmoidal binding curve (Figure 5) with a coherent value of R^2^ (0.9836 and 0.9969, respectively). Nonlinear regression analysis showed that Hill slope (h) is 2.36 for ^99m^Tc, suggesting high positive cooperativity (positive allosteric effect) between ^99m^Tc and MAA. Positive cooperativity (h > 1) also implies that the first protein molecules bind to the MAA with a lower affinity than do subsequent protein molecules, perhaps in a rearrangement of lateral aminoacidic chains exposed on the MAA surface. The Hill coefficient for ^68^Ga is 0.893, a value h < 1 suggests that MAA have multiple different affinity binding sites for gallium. This result is coherent with SEM observations.

From results of the Langmuir isotherms, it was determined that there is a slight difference between the number of atoms of ^99m^Tc and ^68^Ga bound at equilibrium and specifically bound to the HSA that form up a single MAA particle (Table 3). In contrast to what happens for NC [15], MAA show no discrimination between the two radioisotopes, coherent with the absence of binding pockets. This experiment matches with the one of double radiolabelling with ^99m^Tc and ^68^Ga, where no preference is shown from MAA.

HSA-based drugs are the first pharmaceutical in which amino acids satisfy all the requirements without the use of other coligands, but nothing is known about which ones are involved in the binding [24].

The results suggest that MAA behaviour matches with solvent-exposed glutamate and aspartate amino acids, which have been shown to represent binding sites for multivalent cations with low affinity and low cation specificity [25].

In contrast for ^99m^Tc, most of the ^68^Ga PET imaging drugs are labelled via an appropriate bifunctional chelator, such as ^68^Ga-DOTATATE, ^68^Ga-DOTATOC or ^68^Ga-DOTANOC [26,27,28].

Despite efforts to develop suitable chelators for ^68^Ga radiolabelling, full protein-derived drugs without the need of a macrocycle for ^68^Ga labelling could be an interesting development [29,30].

Thus, because protonation and deprotonation modulate the charge state of basic and acidic amino acid side chains (e.g., Lys, Arg, Glu, Asp, His, for which Tc has great affinity [31]) as well as that of the protein termini (carboxy and amino terminus [17]), and their ability for complex formation with a metal, a better understanding of the pH influence could be helpful for developing an efficient labelling procedure. For both methods used, the best pH for radiolabelling is 6, as actually reported in the radiopharmaceutical specifications. With Method 2, the pH modification did not lead to a decrease in the bound fraction, but not as substantially as it does with Method 1. Probably, if MAA are allowed to reorganise the structure, the surfaces exhibit a different amino acid pattern (protonated or not), capable of binding ^99m^Tc. Instead, if the pH is changed after the bond is created, the binding complex breaks down and fails to reform, due to a series of side reactions that change the state of technetium [32]. The behaviour of MAA is completely opposite to that of NC [23], probably because a multitude of different amino acid side chains are exposed on the MAA surface, which, unlike NC, lead to a nondiscriminatory environment. Therefore, the change of pH can break a previously formed complex but cannot interfere in creating a new complex with different amino acid side chains.

Lastly, the competition with DMSA for ^99m^Tc stability bound when using Methods 1 and 2 (Table 7) reveal that the fraction of ^99m^Tc that resists transchelation for MAA is similar to that of HSA for both methods (Table 6). This means that different types of bonds are formed during the labelling procedure: some bonds are more stable and indissoluble by DMSA addition, while others are instead weaker and easily attacked by the chelating agent. It is probable that MAA does not have a binding pocket; thus, locating the metal too close to the solvent [33] facilitates its transchelation. An attempt was made to normalise the value of ^99m^Tc atoms not affected by the transchelation process (Method 2) to the number of HSA and human serum albumin molecules forming a single MAA particle. We obtained only 1.95(2) 1 × 10^−4 99m^Tc atoms that are tightly bound to HSA (about 1 atom/5000) and for MAA value (1.44(2) 1 × 10^7^). It was then normalised to the number of human serum albumin molecules forming a single MAA particle, obtaining (8.29(2) 1 × 10^−5^), which is even lower than that of HSA (about 1 atom/10,000 tightly bound) at SPC condition. A value sharply different from NC (46.4(2)) and from human serum albumin forming a single NC particle (2.1(2)) was observed [23].

## 5. Conclusions

Improved knowledge of the labelling mechanism of HAS-based radiopharmaceuticals is essential to predict its behaviour with different isotopes. The experimental results were generally consistent with each other. Radiolabelling is possible with high efficiency and with high speed for both ^99m^Tc (Bmax: 46.32, h: 2.36) and ^68^Ga (Bmax: 44.54, h: 0.893). Due to the absence of competition for the same binding sites, dual labelling with ^99m^Tc and ^68^Ga is also possible without any problems. Our experience confirmed that pH is an essential parameter influencing technetium labelling. Therefore, to provide the best condition for the repositioning of Pulmocis^®^, we will investigate the role of pH on ^68^Ga labelling. Based on our results, we assume that MAA binds isotopes on its surface, resulting in bonds that are weaker and less specific than another well-known HSA-based drug, NC. Further studies will be conducted that focus on radiolabelling under physiological pH variations and a hypoxic condition, which may modify the binding properties of the amino acids.

## Figures and Tables

**Table 1:** Activity distribution in Pulmocis preparation, shown as a percentage (Mean (SD)).

Bound Fraction	%	Bound Fraction	%
^99m^Tc-MAA	95.13(2)	^68^Ga-MAA	83.22(3)
^99m^Tc-HSA	4.09(2)	^68^Ga-HSA	15.66(3)
Free ^99m^Tc	0.78(2)	Free ^68^Ga	1.11(3)

**Table 2:** Size distribution showed a high percentage of particles with a dimension between 43 and 51 μm (Mean (SD)).

Diameter (μm)	%
<10	1.1(2)
10–20	1.6(2)
20–30	1.2(2)
30–43	0.0(2)
43–51	95.8(2)

**Table 3:** Number of ^99m^Tc and ^68^Ga atoms bound to a single MAA particle and then normalised to the human serum albumin molecules contained in each macroaggregate particle (Mean (SD)).

Isotopes Bound to MAA Particles	Atoms	Isotopes Bound to HSA Molecules Forming a Single MAA Particle	Atoms
^99m^Tc at equilibrium	1.52(1) 1 × 10^10^	^99m^Tc bound at equilibrium	4.7(1)
^68^Ga at equilibrium	1.58(1) 1 × 10^10^	^68^Ga bound at equilibrium	4.9(1)
^99m^Tc specifically bound	1.08(1) 1 × 10^10^	^99m^Tc specifically bound	3.4(1)
^68^Ga specifically bound	9.03(1) 1 × 10^9^	^68^Ga specifically bound	2.8(1)

**Table 4:** ^99m^Tc and ^68^Ga amount bound/unbound to MAA, reported as molar fraction (Mean (SD)).

	^99m^Tc Atoms	^68^Ga Atoms	^99m^Tc Atoms	^68^Ga Atoms
	After 1 h		After 3 h	
Bound molar fraction	4.26(1) 1 × 10^9^	1.56(1) 1 × 10^10^	1.48(1) 1 × 10^10^	1.55(2) 1 × 10^9^
Unbound molar fraction	1.46(1) 1 × 10^9^	2.92(1) 1 × 10^8^	8.98(1) 1 × 10^8^	3.34(2) 1 × 10^9^

**Table 5:** Molar fraction of ^99m^Tc amount bound/unbound to MAA depending on pH values (Mean (SD)).

	Method 1		Method 2	
pH	Bound Molar Fraction	Unbound Molar Fraction	Bound Molar Fraction	Unbound Molar Fraction
10	0.019(1)	0.982(1)	0.674(1)	0.326(1)
6	0.772(1)	0.229(1)	0.709(1)	0.291(1)
3	0.395(1)	0.605(1)	0.702(1)	0.298(1)

**Table 6:** Amount of ^99m^Tc atoms bound to HSA or to DMSA reported as molar fraction (Mean (SD)).

	[^99m^Tc]Tc-HSA Molar Fraction	[^99m^Tc]Tc-DMSA Molar Fraction
Method 1	0.013(1)	0.087(1)
Method 2	0.071(1)	0.029(1)

**Table 7:** Amount of ^99m^Tc atoms bound to MAA or to DMSA reported as molar fraction (Mean (SD)).

	[^99m^Tc]Tc-MAA Molar Fraction	[^99m^Tc]Tc-DMSA Molar Fraction
Method 1	0.029(1)	0.070(1)
Method 2	0.079(1)	0.021(1)

## Data Availability

All data generated or analysed during this study are included in this published article.

## Sample Availability

Samples are available from the authors.
